# Supplementary material for: Avoiding organelle mutational meltdown across eukaryotes with or without a germline bottleneck
Source: PLoS Biol. 2021 Apr 23;19(4):e3001153. doi: 10.1371/journal.pbio.3001153 (PMC8064548; doi:10.1371/journal.pbio.3001153)
Supplement: S1 Text — Stochastic modelling of physical and genetic oDNA dynamics at and between cell divisions and summary of existing data on oDNA during development. oDNA, organelle DNA. (PDF) [file pbio.3001153.s001.pdf]

Avoiding organelle mutational meltdown across eukaryotes with or without a germline bottleneck: S1 Text

Internal oDNA dynamics – general approach

We will consider cells with a population of W wildtype and M mutant oDNA molecules. Several analytical approaches are available to describe the dynamics of these populations. We will use a framework of stochastic population processes [1] for a unified picture of the continuous-time dynamics of oDNA between sampling events (for example, cell divisions). Sampling and reamplifications events will be modelled with the hypergeometric distribution and Polya urn respectively, as described below.

Between sampling events, we consider the processes that affect these populations (replication, degradation, and so on) as Poisson processes. We are interested in heteroplasmy level $h = M/(W + M)$ and its statistics $E(h)$, $V(h)$, and $V'(h) = V(h)/(E(h)(1 - E(h)))$. We also use $N = W + M$, the total oDNA copy number.

These systems can sometimes be solved analytically by using generating functions, but feedback control and other complications challenge this approach. Instead we will use a linear noise approximation and expand the corresponding master equation describing the system to obtain a Fokker-Planck equation describing the time evolution of the state of the system. We then use Itō's formula to change variables and extract a Fokker-Planck equation for heteroplasmy level, from which we then extract the diffusion part describing the variance behaviour.

To capture compartmentalisation, we generally consider four oDNA types W_f (wildtype fused), M_f (mutant fused), W_s (wildtype single), M_s (mutant single). To use the definitions above we have $W = W_f + W_s$ and $M = M_f + M_s$. If we write the state of the system as $\mathcal{X} = (W_f, M_f, W_s, M_s)$, the general set of processes we consider are:

Reactants	Products	Rate (f_i)	Stoichiometry $(\underline{\underline{\mathbf{S}}}^T)_i$	Description	
W_f	$W_f + W_f$	$W_f(\lambda+\delta)(1-\alpha N)$	$(1,0,0,0)$	Fused replication (advantage δ)	(S1)
M_f	$M_f + M_f$	$M_f\lambda(1-\alpha N)$	$(0,1,0,0)$	Fused replication	(S2)
$n_{df}W_f$	$\emptyset$	$W_f\nu_f/n_{df}$	$(-1,0,0,0)$	Fused degradation of n_{df} molecules	(S3)
$n_{df}M_f$	$\emptyset$	$M_f\nu_f/n_{df}$	$(0,-1,0,0)$	Fused degradation of n_{df} molecules	(S4)
W_s	$W_s + W_s$	$W_s\beta_1(\lambda+\delta)(1-\alpha N)$	$(0,0,0,1)$	Single $\rightarrow$ two singles (advantage δ)	(S5)
M_s	$M_s + M_s$	$M_s\beta_1(\lambda)(1-\alpha N)$	$(0,0,0,1)$	Single $\rightarrow$ two singles	(S6)
W_s	$W_f + W_f$	$W_s(1-\beta_1)(\lambda+\delta)(1-\alpha N)$	$(2,0,-1,0)$	Single $\rightarrow$ two fused (advantage δ)	(S7)
M_s	$M_f + M_f$	$M_s(1-\beta_1)\lambda(1-\alpha N)$	$(0,2,0,-1)$	Single $\rightarrow$ two fused	(S8)
n_dW_s	$\emptyset$	$W_s\nu/n_d$	$(0,0,-1,0)$	Degradation of n_d single molecules	(S9)
n_dM_s	$\emptyset$	$M_s\nu/n_d$	$(0,0,0,-1)$	Degradation of n_d single molecules	(S10)
$W_s + W_s$	$W_f + W_f$	$\frac{1}{2}W_s(W_s-1)\alpha_f$	$(2,0,-2,0)$	Two singles fusing	(S11)
$W_f + W_s$	$W_f + W_f$	$W_fW_s\alpha_f$	$(1,0,-1,0)$	Single fusing to fused	(S12)
$M_s + M_s$	$M_f + M_f$	$\frac{1}{2}M_s(M_s-1)\alpha_f$	$(0,2,0,-2)$	Two singles fusing	(S13)
$M_f + M_s$	$M_f + M_f$	$M_fM_s\alpha_f$	$(0,1,0,-1)$	Single fusing to fused	(S14)
W_f	W_s	$W_f\beta_2\alpha_s$	$(-1,0,1,0)$	Single molecule fragmentation	(S15)
M_f	M_s	$M_f\beta_2\alpha_s$	$(0,-1,0,1)$	Single molecule fragmentation	(S16)
$W_f + W_f$	$W_s + W_s$	$\frac{1}{2}W_f(W_f-1)(1-\beta_2)\alpha_s$	$(-2,0,2,0)$	Double fragmentation	(S17)
$M_f + M_f$	$M_s + M_s$	$\frac{1}{2}M_f(M_f-1)(1-\beta_2)\alpha_s$	$(0,-2,0,2)$	Double fragmentation	(S18)
$W_f + M_f$	$W_s + M_s$	$W_fM_f(1-\beta_2)\alpha_s$	$(-1,-1,1,1)$	Double fragmentation	(S19)
$W_f + M_s$	$W_f + M_f$	$W_fM_s\alpha_f$	$(0,1,0,-1)$	Single fusing to fused	(S20)
$W_s + M_f$	$W_f + M_f$	$W_sM_f\alpha_f$	$(1,0,-1,0)$	Single fusing to fused	(S21)
$W_s + M_s$	$W_f + M_f$	$W_sM_s\alpha_f$	$(1,1,-1,-1)$	Two singles fusing	(S22)
$W_f + M_f$	$M_f + M_f$	$W_fM_f\kappa$	$(-1,1,0,0)$	Gene conversion	(S23)
$W_f + M_f$	$W_f + W_f$	$W_fM_f(\kappa+\epsilon)$	$(1,-1,0,0)$	Gene conversion (bias ϵ)	(S24)

Eqns. S1-S10 are genetic processes, corresponding to the replication and degradation of oDNAs within their compartments. Eqns. S11-S22 are physical processes, corresponding to fission and fusion changing the compartment of a contained oDNA. Eqns. S23-S24 are recombination processes, now restricted to the fused compartment.

In coarse-graining a real mitochondrial population into 'fused' and 'singleton' states, several alternative choices have to be made. We encode these choices with parameters β_1 and β_2. Eqns. S5-S6 model a situation where oDNA replication is followed by mitochondrial division, so that the replication of a singleton oDNA leads to the production of two more singletons. By contrast, Eqns. S7-S8 model replication of a singleton within a compartment, with both resulting oDNAs then considered as existing within a fused compartment.

Eqns. S15-S16 assume that fragmentation occurs via a singleton 'budding off' from a fused compartment that remains fused. By contrast, Eqns. S17-S19 assume that fused states are small, so that fragmentation leads to two singletons ('kiss and run').

We will show that neither of these choices affects heteroplasmy statistics.

Different parameter values can be made within this 'umbrella' model to deal with more specific subproblems. Setting $\delta = \epsilon = 0$ removes any selective difference between W and M, so mean heteroplasmy level remains constant. Setting $\nu_f = 0$ prevents degradation of oDNAs in a fused state; setting $\nu_f = \nu$ removes degradation dependence on fission-fusion balance. Organelle dynamics can be neglected by setting $\nu_f = \nu, n_{df} = n_d, \alpha_s = 0, W_s = M_s = 0$ and considering only the dynamics of W_f and M_f (hence retaining only Eqns. S1-S4 and S23-S24). The interpretation of this choice is that all oDNAs can meet and interact within one large connected compartment.

Analysis

Eqns. S1-S24 provide the elements of the stoichiometric matrix $\underline{\underline{\mathbf{S}}}$ and the rate vector $\underline{\mathbf{f}}$ describing the system. We first use the Kramers-Moyal expansion [2, 3] to extract a Fokker-Planck equation for the state of the system.

$$\frac{\partial P(\mathcal{X},t)}{\partial t} = -\sum_{i=1}^{n} \frac{\partial}{\partial \mathcal{X}_i}(A_i(\mathcal{X})P(\mathcal{X},t)) + \frac{1}{2}\sum_{i,j=1}^{N} \frac{\partial^2}{\partial \mathcal{X}_i \partial \mathcal{X}_j}(B_{ij}(\mathcal{X})P(\mathcal{X},t)) \quad \text{(S25)}$$

where

$$\underline{\mathbf{A}} = \underline{\underline{\mathbf{S}}} \cdot \underline{\mathbf{f}} \quad \text{(S26)}$$

$$\underline{\underline{\mathbf{B}}} = \underline{\underline{\mathbf{S}}} \cdot \underline{\underline{\text{diag}}}(\underline{\mathbf{f}}) \cdot \underline{\underline{\mathbf{S}}}^T. \quad \text{(S27)}$$

$\underline{\mathbf{A}}$ and $\underline{\underline{\mathbf{B}}}$ can be written down straightforwardly from $\underline{\underline{\mathbf{S}}}$ and $\underline{\mathbf{f}}$ (given in Eqns. S1-S24), though their large forms prevent their reproduction here. A Mathematica notebook and accompanying PDF documents following this derivation are available at the Github project repository (see Data Statement).

Ref. [2] shows that, using Itō's formula, a corresponding Fokker-Planck equation can be written down for a function h of the random variables under consideration:

$$\frac{\partial P(h,t)}{\partial t} = -\frac{\partial}{\partial h}(\tilde{A}(h)P(h,t)) + \frac{1}{2}\frac{\partial^2}{\partial h^2}(\tilde{B}(h)P(h,t)) \quad \text{(S28)}$$

where

$$\tilde{A} = (\underline{\nabla} h)^T \cdot \underline{\mathbf{A}} + \frac{1}{2}\text{Tr}(\underline{\underline{\mathbf{B} \cdot \mathbf{H}}} h) \quad \text{(S29)}$$

$$\tilde{B} = (\underline{\nabla} h)^T \cdot \underline{\underline{\mathbf{B}}} \cdot (\underline{\nabla} h), \quad \text{(S30)}$$

and $\underline{\underline{\mathbf{H}}}$ is the Hessian matrix and the grad operator takes derivatives with respect to the original random variables $\{W_f, M_f, W_s, M_s\}$:

$$\underline{\nabla} = \left(\frac{\partial}{\partial_{W_f}}, \frac{\partial}{\partial_{M_f}}, \frac{\partial}{\partial_{W_s}}, \frac{\partial}{\partial_{M_s}}\right) \quad \text{(S31)}$$

$$\underline{\underline{\mathbf{H}}} = \begin{bmatrix} \frac{\partial^2}{\partial W_f^2} & \dots & \frac{\partial^2}{\partial W_f \partial M_s} \\ \dots & \dots & \dots \\ \frac{\partial^2}{\partial W_f \partial M_s} & \dots & \frac{\partial^2}{\partial M_s^2} \end{bmatrix}. \quad \text{(S32)}$$

Finally, ODEs for the time behaviour of the moments can be extracted from the Fokker-Planck equation (Eqn. S28),

$$\frac{dE(h)}{dt} = \tilde{A}, \quad \text{(S33)}$$

$$\frac{dV(h)}{dt} = 2\tilde{A}V(h) + \tilde{B}. \quad \text{(S34)}$$

No selection

In the case where $\delta = \epsilon = 0$, no selective pressure acts to change heteroplasmy level over time. The drift term in Eqn. S28 then vanishes, and we are interested in the diffusion behaviour of h, the time coefficient of which is immediately given by $\tilde{B}$ in Eqn. S28. From the form of the matrix B, and writing $W_f = (1-f)(1-h)n, M_f = (1-f)hn, W_s = f(1-h)n, M_s = fhn$, we obtain

$$\tilde{B} = \frac{h(1-h)}{n}\left(2(1-f)^2 n\kappa + (1-\alpha N)\lambda + f n_d \nu + (1-f) n_{df} \nu_f\right) \quad \text{(S35)}$$

where it will be seen that β_1 and β_2 play no role in this expression. We thus obtain

$$V'(h) = \left(2\kappa + \frac{(1-\alpha N)\lambda + n_d\nu)}{N}\right) t, \text{ no compartments} \quad \text{(S36)}$$

$$V'(h) = \left(2\kappa(1-f)^2 + \frac{(1-\alpha N)\lambda + f n_d \nu)}{N}\right) t, \text{ with compartments.} \quad \text{(S37)}$$

To see that this aligns with the corresponding $2\nu f/N$ expression from Ref. [2], we first estimate the time behaviour of $N = W_f + M_f + W_s + M_s$ using Itō's formula (as we did for h in Eqn. S28 above). We find

$$\frac{dN}{dt} = N(\delta + \lambda - \nu_f + (\nu_f - \nu)f - \alpha\delta N - \alpha\lambda N + \delta h(\alpha N - 1)) \quad \text{(S38)}$$

$$= N(\lambda - \nu f - \alpha\lambda N) \quad \text{(S39)}$$

$$N_{ss} = \frac{\lambda - \nu f}{\alpha\lambda} \quad \text{(S40)}$$

$$\lambda = \frac{\nu f}{1 - \alpha N_{ss}} \quad \text{(S41)}$$

where the second line follows for neutral genetics ($\delta = dh/dt = 0$), equilibrated mitochondrial dynamics ($df/dt = 0$), and $\nu_f = 0$, and N_{ss} is the value of N for which $dN/dt = 0$.

Then if $N = N_{ss}$:

$$\frac{(1-\alpha N)\lambda + f n_d \nu}{N} = \frac{\nu f + f n_d \nu}{N} = \frac{(n_d+1)\nu f}{N}, \quad \text{(S42)}$$

and if $n_d = 1$ (as in Ref. [2]) we recover $2\nu f/N$.

Selection

In the case of nonzero δ and/or ϵ, mean heteroplasmy level can change over time. The drift behaviour of Eqn. S28 is given by

$$\frac{dE(h)}{dt} = \tilde{A} \quad \text{(S43)}$$
$$= \frac{1}{N} h(1-h)(\delta(N-1)(\alpha N - 1) - \epsilon N^2 (1-f)^2), \quad \text{(S44)}$$

where we have written $N = W_f + M_f + W_s + M_s$ and $f = (W_s + M_s)/N$. In general, N and f will themselves be varying with time (applying Itō's formula as above yields the corresponding Fokker-Planck equations), leading to a system of three coupled nonlinear ODEs. We can make progress by assuming that genetic changes occur more slowly than physical changes. This is not an unreasonable assumption for organelles, where, for example, mitochondrial fission and fusion occur on the timescale of minutes [4], while mtDNA replication and degradation has a timescale of hours or days [5].

This assumption allows us to treat N and f as equilibrated constants in Eqn S44 and thus to find

$$E(h) = \frac{1}{1 + \left(\frac{1}{h_0} - 1\right) \exp((-\gamma_1 + \gamma_2)t/N)}, \quad \text{(S45)}$$

where $\gamma_1 = \delta(\alpha N - 1)(N-1)$ and $\gamma_2 = \epsilon N^2 (1-f)^2$ are rescaled selective coefficients due to replication and biased gene conversion respectively.

The expression for the variance of h is more involved

$$\frac{dV(h)}{dt} = 2\tilde{A}V(h) + \tilde{B} \quad \text{(S46)}$$
$$= \frac{h(1-h)}{N} \left((1-f)^2 N(\epsilon + 2\kappa) + \lambda - \alpha\lambda N + \nu n_d f - \delta(\alpha N - 1)E(h) \right.$$
$$\left. -2(-\gamma_1 + \gamma_2)V(h)\right). \quad \text{(S47)}$$

Substituting Eqn. S45 into Eqn. S47 and solving we obtain

$$V(h) = \left(e^{-2h_0}\left(e^{2h_0+bt}(h_0-1)(\phi-2\rho) - e^{2h_0}h_0(3\phi-2\rho) - \exp\left(\frac{2h_0}{(1-h_0)e^{bt}+h_0} + bt\right)(h_0-1)(\phi(2h_0+1)-2\rho)\right.\right.$$
$$\left.\left. + \exp\left(\frac{2h_0}{(1-h_0)e^{bt}+h_0}\right) h_0((2h_0+1)\phi - 2\rho)\right)\right) / (4(\gamma_1-\gamma_2)(e^{bt}(h_0-1)-h_0)) \quad \text{(S48)}$$

where $\rho = (1-f)^2 N(\epsilon + 2\kappa) + \lambda - \alpha\lambda N + \nu n_d f$, $\phi = \delta(\alpha n - 1)$, and $b = (-\gamma_1 - \gamma_2)/n$.

If gene conversion is ignored ($\kappa = \epsilon = 0$), some further approximations and substitutions allow us to simplify these forms. If we assume that N is large enough so that $N - 1 \simeq N$, then

$$E(h) = \frac{1}{1+e^{-\rho t}} \quad \text{(S49)}$$

$$V(h) = \frac{\exp\left(\frac{-2}{1+e^{\rho t}}\right)}{4(e^{\rho t}+1)N\rho}\left(4e\nu f + 4\nu f e^{\rho t+1} + \exp\left(\frac{2}{e^{\rho t}+1}+\rho t\right)(\rho - 4\nu f) - \exp\left(\frac{2}{e^{\rho t}+1}\right)(\rho + 4\nu f)\right) \quad \text{(S50)}$$

where $\rho = \delta(\alpha N - 1)$. These expressions are useful in comparison with experimental heteroplasmy observations as νf, ρ, and N are the only variable combinations required to be independently identified.

All sources of variance

We employ a linear noise approximation and write:

$$\begin{aligned} V'(h) &= \sum_{divisions\, i} V'_{division}(n_{1,i}) \\ &+ \sum_{cell\, cycles\, i} (V'_{turnover}(n_i, \tau_i, \nu_i) + V'_{conversion}(\kappa_i, \tau_i)) \\ &+ \sum_{subsamples\, i} V'_{sampling}(n_{1,i}, n_{2,i}) + \sum_{amplifications\, i} V'_{amplification}(n_{1,i}, n_{2,i}) \end{aligned} \quad \text{(S51)}$$

Individual oDNA dynamics

In the case of no selection, $V'_{turnover}(n, \tau, \nu)$ and $V'_{conversion}(\kappa, \tau)$ can be read off from Eqn. S37 above. In the case of selection, as $E(h)$ changes over time, there is ambiguity in how to define $V'(h)$: should $V(h)$ be normalised by the constant $h_0(1-h_0)$, the time-varying $E(h)(1-E(h))$, or otherwise? For consistency with existing models [6], we recommend the former option. The expression for $V(h)$ from Eqn. S48 can be used and normalised as desired.

Subsampling and cell divisions (without replacement)

Let sampling event i begin with a population of size n_1, containing m mutants, and sample a new population of size n_2. If this sampling takes place without replacement, the probability of sampling k mutants is given by the hypergeometric distribution

$$P(k; n_1, n_2, m) = \frac{\binom{m}{k}\binom{n_1-m}{n_2-k}}{\binom{n_1}{n_2}}, \quad \text{(S52)}$$

which has variance $V(k) = n_2(m/n_1)(1-m/n_1)\left(\frac{n_1-n_2}{n_1-1}\right)$ and so

$$V'_{sampling}(n_1, n_2) = \frac{1}{n_2}\left(\frac{n_1-n_2}{n_1-1}\right). \quad \text{(S53)}$$

Hence if cell division i is a sampling event with $n_2 = n_1/2$,

$$V'_{division\, i} = \frac{1}{n_1-1} \quad \text{(S54)}$$

If the units of sampling are clusters of size n_c rather than individual oDNA molecules, the variance effect depends on the makeup of these clusters. If clusters can be heteroplasmic, and are themselves sampled from

the original population, the variance effect is unchanged [7]. If oDNAs are clustered and sampled in homoplasmic clusters, the quantities in Eqn. S52 are scaled accordingly:

$$P(k'; n_1', n_2', m') = \frac{\binom{m'}{k'}\binom{n_1'-m'}{n_2'-k'}}{\binom{n_1'}{n_2'}}, \tag{S55}$$

where primed quantities $x' = x/n_c$. As the quantities here must be integers, some errors due to rounding are possible, corresponding physically to ignoring left-over oDNA molecules that do not fit into a cluster. The corresponding variance is $V(k') = n_2/n_c(m/n_1)(1-m/n_1)\left(\frac{(n_1-n_2)/n_c}{n_1/n_c-1}\right)$, and as $h = k'n_c/n_2$,

$$V'_{sampling}(n_1, n_2) = \frac{n_c}{n_2}\left(\frac{n_1-n_2}{n_1-n_c}\right). \tag{S56}$$

Now for a division with $n_2 = n_1/2$,

$$V'_{division} = \frac{n_c}{n_1-n_c}. \tag{S57}$$

Subsampling and cell divisions (with replacement)

For completeness we include the alternative, where oDNAs are sampled *with* replacement. Then the probability of sampling k mutants is given by the binomial distribution

$$P(k; n_1, n_2, m) = \binom{n_2}{k}(m/n_1)^k(1-m/n_1)^{n_2-k} \tag{S58}$$

which has variance $V(k) = n_2(m/n_1)(1-m/n_1)$ and so

$$V'_{sampling} = \frac{1}{n_2} \tag{S59}$$

Hence if cell division i is a sampling event with $n_2 = n_1/2$,

$$V'_{division i} = \frac{2}{n_1}. \tag{S60}$$

In the case of homoplasmic oDNA clusters of size n_c, the same argument as above holds, and the variance contributions are

$$V'_{sampling} = \frac{n_c}{n_2} \tag{S61}$$

$$V'_{division} = \frac{2n_c}{n_1}. \tag{S62}$$

Amplification

Let reamplification event i begin with a population of size n_1 and end with a population of size n_2. We model this process as a Pólya urn, initially containing w wildtype and m mutant oDNAs. Each iteration, an oDNA is drawn and duplicated, and both oDNAs are returned to the urn. After $n_2 - n_1$ iterations, each increasing the population size by one, the probability of k mutant oDNAs is given by the beta-binomial distribution

$$P(k|n_2-n_1, m, w) = \frac{\Gamma(n_2-n_1+1)}{\Gamma(k+1)\Gamma(n_2-n_1-k+1)}\frac{\Gamma(k+m)\Gamma(n_2-n_1-k+w)}{\Gamma(n_2-n_1+m+w)}\frac{\Gamma(w+m)}{\Gamma(w)\Gamma(m)} \tag{S63}$$

with variance

$$V(k) = \frac{(n_2 - n_1)wm(w+m+n_2-n_1)}{((w+m)^2(w+m+1))} \quad \text{(S64)}$$

$$= \frac{(n_2 - n_1)(1 - m/n_1)(m/n_1)n_2}{(n_1+1)} \quad \text{(S65)}$$

so

$$V'_{reamplification}(h) = \frac{(n_2 - n_1)}{n_2(n_1+1)} \quad \text{(S66)}$$

and if we consider reamplification to the level before a symmetric cell division, $n_2 = 2n_1$ and

$$V'_{reamplification}(h) = \frac{1}{2(n_1+1)}. \quad \text{(S67)}$$

Non-interacting organelles

The processes in Eqns. S1-S24 assume that all oDNAs in a cell can, at least in principle, interact. This picture corresponds more to a mitochondrial system, where the 'chondriome' is fluid and individual mitochondria (inasmuch as individual organelles exist) can share content. By contrast, plastids rarely fuse and the set of oDNAs in a single plastid behaves more as an isolated population, with the cell consisting of many individual isolated populations.

Let h_i be the heteroplasmy level in organelle i, and h as before be the heteroplasmy level of the whole cell. Assuming the cell contains n_{org} organelles each containing similar copy numbers of oDNA,

$$h = \frac{1}{n_{org}} \sum_{i=1}^{n_{org}} h_i \quad \text{(S68)}$$

$$E(h) = \frac{1}{n_{org}} \sum_{i=1}^{n_{org}} E(h_i) \quad \text{(S69)}$$

$$V(h) = \frac{1}{n_{org}^2} \sum_{i=1}^{n_{org}} V(h_i) \quad \text{(S70)}$$

$$V'(h) = V(h)/(E(h)(1-E(h))) \quad \text{(S71)}$$

For a single chondriome, $n_{org} = 1$ and $V(h) = V(h_1)$.

For multiple independent organelles, we proceed by splitting the variance behaviour of each organelle's population into a population-size-dependent part a/N_i and a population-size-independent part b:

$$V(h_i) = E(h_i)(1-E(h_i))\left(\frac{a}{N_i} + b\right)t, \quad \text{(S72)}$$

where N_i is the copy number of oDNAs in organelle i.

Let all organelles have equal properties, so that $N_i = N/n_{org}$ and $E(h_i) = \bar{h}$. Then

$$V'(h) = \frac{1}{n_{org}^2}\sum V(h_i)/(\frac{1}{n_{org}}\sum E(h_i)\left(1-\frac{1}{n_{org}}\sum E(h_i)\right) \tag{S73}$$

$$= \frac{\frac{1}{n_{org}^2} n_{org}\left(\frac{a}{N/n_{org}}+b\right) t\bar{h}(1-\bar{h})}{\bar{h}(1-\bar{h})} \tag{S74}$$

$$= \left(\frac{a}{N}+\frac{b}{n_{org}}\right)t \tag{S75}$$

So that the contribution from population-size-dependent processes remains the same as the continuous case, but the contribution from population-size-independent processes is limited by n_{org}^{-1}. This result reflects the fact that gene conversion is more limited in a set of small independent genetic 'pools' than in one large interacting system.

Total variance

In total

$$\begin{aligned} V'(h) &= \sum_{divisions\,i} V'_{division}(n_{1,i}) \\ &+ \sum_{cell\,cycles\,i} (V'_{turnover}(n_i,\tau_i,\nu_i) + V'_{conversion}(\kappa_i,\tau_i)) \\ &+ \sum_{subsamples\,i} V'_{sampling}(n_{1,i},n_{2,i}) + \sum_{amplifications\,i} V'_{amplification}(n_{1,i},n_{2,i}) \quad \text{(S76)} \\ &= \sum_{divisions\,i}\frac{n_{c,i}}{n_{1,i}-n_{c,i}} + \sum_{cell\,cycles\,i} 2\left(\frac{\nu_i\tau_i}{n_i}+\kappa_i\tau_i\right) + \sum_{subsamples\,i}\frac{n_{c,i}}{n_{2,i}}\left(\frac{n_{1,i}-n_{2,i}}{n_{1,i}-n_{c,i}}\right) \\ &+ \sum_{amplifications\,i}\frac{n_{2,i}-n_{1,i}}{n_{2,i}(n_{1,i}+1)} \quad \text{(S77)} \end{aligned}$$

In other words, recombination is the only strategy that is not scaled by population size

In most cases where population size is much greater than n_c we can approximate

$$\begin{aligned} V'(h) &\simeq \sum_{divisions\,i}\frac{n_{c,i}}{n_{1,i}} + \sum_{cell\,cycles\,i} 2\left(\frac{\nu_i\tau_i}{n_i}+\kappa_i\tau_i\right) + \sum_{subsamples\,i} n_{c,i}\left(\frac{1}{n_{2,i}}-\frac{1}{n_{1,i}}\right) \\ &+ \sum_{amplifications\,i}\left(\frac{1}{n_{1,i}}-\frac{1}{n_{2,i}}\right) \quad \text{(S78)} \end{aligned}$$

In S1 Fig we show time series predictions of $V'(h)$ from Eqn. S78 with stochastic simulations for several different mechanisms, illustrating the generally good agreement between theory and simulation.

Repeated cell cycles

Let N_i be copy number after the ith amplification and division. Each cell cycle, copy number changes from N_i to αN_i, then is depleted by division to $\alpha N_i/2 = N_{i+1}$, so that $N_i = (\alpha/2)^i N_0$. A change in copy number from N_0 to N_k over k divisions implies that

$$\alpha = 2(N_k/N_0)^{1/k}. \tag{S79}$$

Hence, for cell cycle i,

$$V'_{amplification}(h) = \left(\frac{1}{n_1} - \frac{1}{n_2}\right) \quad \text{(S80)}$$
$$= \left(\frac{1}{N_i} - \frac{1}{\alpha N_i}\right) \quad \text{(S81)}$$
$$= \frac{(2/\alpha)^i(\alpha - 1)}{\alpha N_0} \quad \text{(S82)}$$
$$V'_{division}(h) = \frac{n_c}{n_1} \quad \text{(S83)}$$
$$= \frac{n_c}{\alpha N_i} \quad \text{(S84)}$$
$$= \frac{(2/\alpha)^i n_c}{\alpha N_0} \quad \text{(S85)}$$

for a total of

$$V'_i(h) = \frac{(2/\alpha)^i(\alpha + n_c - 1)}{\alpha N_0} \quad \text{(S86)}$$

The total variance increase after the kth cell division is then

$$V'(h) = \sum_{i=0}^{k-1} V'_i(h) \quad \text{(S87)}$$
$$= \frac{\alpha + n_c - 1}{\alpha N_0} \sum_{i=0}^{k-1} (2/\alpha)^i \quad \text{(S88)}$$
$$= \frac{\alpha + n_c - 1}{\alpha N_0} \frac{(2/\alpha)^k - 1}{(2/\alpha) - 1} \quad \text{(S89)}$$

Physical behaviour at cell divisions

We used physical simulation to investigate how the physical arrangement of organelles influences their inheritance at cell divisions. We assume a spherical cell of unit radius, and simulate N physical agents within this cell. These agents are subjected to diffusive motion and spread through the cell over time. In the non-interacting case, this motion spreads the agents randomly through the cell. In the interacting case, each agent experiences a repulsive interaction from other agents, scaled by the squared distance between them.

Specifically, if $\underline{\mathbf{x_i}}$ is the position of agent i and $\underline{\mathbf{R}}$ is a vector with components $R_i \sim U(-d, d)$, we write

$$\underline{\mathbf{\Delta r_i}} = \underline{\mathbf{R}} \quad \text{(S90)}$$
$$\underline{\mathbf{\Delta r_i^I}} = \sum_{j \neq i} \frac{\underline{\mathbf{x_i}} - \underline{\mathbf{x_j}}}{|\underline{\mathbf{x_i}} - \underline{\mathbf{x_j}}|^2}. \quad \text{(S91)}$$

then in the non-interacting case,

$$\underline{\mathbf{x_i}} \rightarrow \underline{\mathbf{x_i}} + \underline{\mathbf{\Delta r_i}}, \; \underline{\mathbf{x_i}} + \underline{\mathbf{\Delta r_i}} \in B(1) \quad \text{(S92)}$$
$$\underline{\mathbf{x_i}} \rightarrow \underline{\mathbf{x_i}}, \text{ otherwise} \quad \text{(S93)}$$

and in the interacting case,

$$\begin{aligned}
\underline{\mathbf{x_i}} &\rightarrow \underline{\mathbf{x_i}} + \underline{\mathbf{\Delta r_i}},\ \underline{\mathbf{x_i}} + \underline{\mathbf{\Delta r_i}} \in B(1) && \text{(S94)}\\
\underline{\mathbf{x_i}} &\rightarrow \underline{\mathbf{x_i}},\ \text{otherwise} && \text{(S95)}\\
\underline{\mathbf{x_i}} &\rightarrow \underline{\mathbf{x_i}} + \frac{\underline{\mathbf{\Delta r_i^I}}}{|\underline{\mathbf{\Delta r_i^I}}|},\ \frac{\underline{\mathbf{\Delta r_i^I}}}{|\underline{\mathbf{\Delta r_i^I}}|} \in B(1) && \text{(S96)}\\
\underline{\mathbf{x_i}} &\rightarrow \underline{\mathbf{x_i}},\ \text{otherwise.} && \text{(S97)}
\end{aligned}$$

These processes were chosen for algorithmic simplicity and not to reproduce realistic microscopic dynamics. The (intuitive) long-term outcomes are a random distribution of agents in the non-interacting case, and a more even spread of agents in the interacting case (S3 Fig).

We labelled half of the agents as wildtype oDNA and half as mutant, and asked how their physical arrangement influence the genetic properties of cell division. Dividing the unit ball by the $x = 0$ plane, we recorded the variance in copy number and heteroplasmy level in the resulting daughter cells. We found, as expected, that the non-interacting case yielded binomial partitioning of copy number and the interacting case led to lower copy number variance between daughters (closer to even inheritance) (S3 Fig). However, both cases had the same hypergeometric inheritance pattern of heteroplasmy (S3 Fig).

Physical dynamics of plant mtDNA

Plant mtDNA exists in diverse forms in plant cells, with some mitochondria containing no mtDNA, some molecules containing only a subset of the mitochondrial genome, and others containing the full set. Recombination mixes these physical forms in a 'dynamic syncytium' (or 'madness' [8]).

Partitioning the genome through recombination-mediated splitting, and allowing independent dynamics of these split molecules, provides a means of generating sequence diversity in the cell, and specifically another mechanism for effective gene conversion. To see this, consider the tripartite system $\mathcal{AB} \leftrightarrow \mathcal{A} + \mathcal{B}$ [9], variants of which have been studied in classical analytic and simulation work [10, 11], and which experiments suggest to reflect the system in, for example, *Brassica napus* [12] (although the 'master circle' picture of plant mtDNA structure [13] has been questioned [14]). Say locus $\mathcal{B}$ is polymorphic, so we can have mtDNA haplotypes aB and ab. Then, given two full starting genomes, recombination-mediated splitting can cause

$$\begin{aligned}
aB + aB &\rightarrow 2a + 2B && \text{(S98)}\\
ab + aB &\rightarrow 2a + b + B && \text{(S99)}\\
ab + ab &\rightarrow 2a + 2b. && \text{(S100)}
\end{aligned}$$

Effective 'gene conversion' can follow from $ab + aB \rightarrow 2a + b + B$, if the subgenomic molecules evolve independently. If, for example, the resultant B replicated (and, optionally, the resultant b degrades), then both B molecules recombine with as:

$$
\begin{aligned}
ab &\rightarrow_{\kappa_s} a + b && \text{(S101)}\\
aB &\rightarrow_{\kappa_s} a + B && \text{(S102)}\\
b &\rightarrow_{\nu_s} \emptyset && \text{(S103)}\\
B &\rightarrow_{\lambda_s} B + B && \text{(S104)}\\
a + B &\rightarrow_{\kappa_f} aB && \text{(S105)}\\
a + B &\rightarrow_{\kappa_f} aB && \text{(S106)}\\
& && \text{(S107)}
\end{aligned}
$$

which is effectively $W + M \rightarrow M + M$:

$$
\begin{aligned}
W &\rightarrow W_1 + W_2 && \text{(S108)}\\
M &\rightarrow W_1 + M_2 && \text{(S109)}\\
W_2 &\rightarrow \emptyset && \text{(S110)}\\
M_2 &\rightarrow M_2 + M_2 && \text{(S111)}\\
W_1 + M_2 &\rightarrow M && \text{(S112)}\\
W_1 + M_2 &\rightarrow M. && \text{(S113)}
\end{aligned}
$$

Of course, many other combinations of recombination, replication, and degradation events can occur. If neither b nor B replicate or degrade before recombining with the two a molecules, the overall outcome will be the neutral $W + M \rightarrow W + M$ ($ab \rightarrow a + b, aB \rightarrow a + B, a + b \rightarrow ab, a + B \rightarrow aB$). If both degrade, we have $W + M \rightarrow 2a$ ($ab \rightarrow a + b, aB \rightarrow a + B, b \rightarrow \emptyset, B \rightarrow \emptyset$, leaving $2a$). If b does nothing before recombination, and B degrades, there will be a stoichiometric imbalance: $W + M \rightarrow W + a$ (or $W + M \rightarrow M + a$ in the opposite case). Similarly, if there is a replicative excess, imbalances can arise $W + M \rightarrow W + b$ or $W + M \rightarrow M + B$, or different patterns if more replication or degradation events occur (or if a molecules replicate or degrade). In these instances where a, b, or B molecules remain, there is a risk of selfish proliferation of these reduced elements [10, 11].

The balance between contributions to beneficial segregation and the risk of selfish proliferation is therefore controlled by the relative rates of these processes. Intra-molecular recombination (splitting) should occur reasonably rarely or in limited compartments, to prevent a large interacting pool of reduced molecules. Replication of these reduced molecules should be nonzero but limited, and inter-molecular recombination (merging) should have a comparable rate to the replication and degradation dynamics, to harness the diversity thus generated but prevent further proliferation. Clearly this balance is challenging to strike quantitatively, as seen in plant mitochondria which frequently contain reduced (or no) mtDNA molecules [15], and experience substoichiometric shifting where one initially rare mtDNA type rapidly comes to dominate the cellular population [8, 16].

Transformed heteroplasmy level

It is often convenient to transform heteroplasmy level measurements using

$$\Delta = g(h; h_0) = \log\left(\frac{h(h_0 - 1)}{h_0(h - 1)}\right) \quad \text{(S114)}$$

$$g^{-1}(\Delta; h_0) = \frac{1}{1 + \frac{1-h_0}{h_0} e^{-\Delta}}. \quad \text{(S115)}$$

This sigmoidal transformation accounts for the restricted domain of h (and the fact that changes in h becomes 'harder' as the limits $h = 0$ and $h = 1$ are approached). In general cases we can use $h_0 = \frac{1}{2}$ for a symmetric transformation; when we have measurements of h over time we can take h_0 to be an initial reference measurement and regard $\Delta = \beta t$ as the shift over time induced by selective pressure β.

The theory above deals with the statistics of untransformed heteroplasmy level. To interpret this with respect to transformed heteroplasmy level measurements we wish to estimate the statistics of the function $g(h)$ of a random variable h.

If the moments of h are finite, standard results from Taylor expansions can be used here:

$$E(g(h)) \simeq g(E(h)) + \frac{g''(E(h))}{2} V(h) \quad \text{(S116)}$$

$$V(g(h)) \simeq (g'(E(h)))^2 V(h), \quad \text{(S117)}$$

which immediately give

$$E(g(h)) \simeq \log\left(\frac{E(h,t)}{1 - E(h,t)}\right) + \frac{V(h,t)(2E(h,t) - 1)}{2(E(h,t))^2(E(h,t) - 1)^2} \quad \text{(S118)}$$

$$V(g(h)) \simeq \frac{V(h,t)}{(E(h,t))^2(E(h,t) - 1)^2}. \quad \text{(S119)}$$

We can then use expressions for $E(h,t)$ (Eqn. S45) and $V(h,t)$ (Eqn. S48; but for the comparison with data in the main text we use Eqn. S49 and Eqn. S50 respectively) in these expressions to predict the statistics of transformed heteroplasmy level over time.

mtDNA copy number in germline development

In mice, mtDNA copy number measurements are taken by Refs. [17, 18, 19] (S4 Fig).

In plants, measurements are less complete (S4 Fig). Takanashi et al. find mtDNA copy numbers around 2000 in rice egg cells, compared to around 120 in leaf protoplasts [20]. Kuroiwa finds 300Mb of mtDNA in a 'mitochondrial complex' in the mature egg cell of *Pelargonium zonale* [21]. The absence of *Pelargonium* mtDNA sequence information prevents an immediate readout of copy number, but estimation based on the 737kb genome of a cousin *Geranium maderense* (NCBI KP940515.1), suggests a copy number around 450. Lamppa et al. find 410 mtDNAs per cell in pea embryo, decreasing to around 250 in leaves, 130 in roots, and 140 in shoots [22]. Preuten et al. find around 350 mtDNAs per cell in *Arabidopsis* root tip, compared to 100 or fewer in roots and siliques, and 50 or fewer in stems and flowers [15]. Wang et al. found egg cells from *Arabidopsis*, *Antirrhinum majus*, and *Nicotiana tabacum* to possess 59.0, 42.7, and 73.0 copies of mtDNA on average, respectively [23]. The first (to our knowledge) survey of mtDNA copy number in early plant development was performed by Gao et al. in *Arabidopsis* and *Torenia fournieri*, finding copy number averages of 78.0, 154.2, 33.4 and 103.0 in mature egg cells, late zygotes, early apical, and early basal cells respectively [24]. By contrast with the copy number reduction

seen in mammalian development, these early plant stages thus seem to show, if anything, an amplification of mtDNA.

References

[1] Eric Renshaw. *Stochastic population processes: analysis, approximations, simulations*. OUP Oxford, 2015.

[2] Juvid Aryaman, Charlotte Bowles, Nick S Jones, and Iain G Johnston. Mitochondrial Network State Scales mtDNA Genetic Dynamics. *Genetics*, 212(4):1429–1443, 2019.

[3] Crispin W Gardiner et al. *Handbook of stochastic methods*, volume 3. springer Berlin, 1985.

[4] Gilad Twig, Brigham Hyde, and Orian S Shirihai. Mitochondrial fusion, fission and autophagy as a quality control axis: the bioenergetic view. *Biochimica et Biophysica Acta (BBA)-Bioenergetics*, 1777(9):1092–1097, 2008.

[5] Iain G Johnston and Nick S Jones. Evolution of cell-to-cell variability in stochastic, controlled, heteroplasmic mtDNA populations. *The American Journal of Human Genetics*, 99(5):1150–1162, 2016.

[6] Iain G Johnston. Varied mechanisms and models for the varying mitochondrial bottleneck. *Frontiers in Cell and Developmental Biology*, 7, 2019.

[7] Iain G Johnston, Joerg P Burgstaller, Vitezslav Havlicek, Thomas Kolbe, Thomas Rülicke, Gottfried Brem, Jo Poulton, and Nick S Jones. Stochastic modelling, Bayesian inference, and new in vivo measurements elucidate the debated mtDNA bottleneck mechanism. *Elife*, 4, 2015.

[8] Magdalena Woloszynska. Heteroplasmy and stoichiometric complexity of plant mitochondrial genomes – though this be madness, yet there's method in't. *Journal of experimental botany*, 61(3):657–671, 2010.

[9] Volker Brendel and Lee A Segel. On modes of recombination, replication, and segregation of the higher plant mitochondrial genome. *Journal of theoretical biology*, 125(2):163–176, 1987.

[10] Beatrice Albert, Bernard Godelle, Anne Atlan, Rosine De Paepe, and Pierre Henri Gouyon. Dynamics of plant mitochondrial genome: model of a three-level selection process. *Genetics*, 144(1):369–382, 1996.

[11] Anne Atlan and Denis Couvet. A model simulating the dynamics of plant mitochondrial genomes. *Genetics*, 135(1):213–222, 1993.

[12] Jianmei Chen, Rongzhan Guan, Shengxin Chang, Tongqing Du, Hongsheng Zhang, and Han Xing. Sub-stoichiometrically different mitotypes coexist in mitochondrial genomes of *Brassica napus L.* *PLoS one*, 6(3):e17662, 2011.

[13] David M Lonsdale, Tony P Hodge, and Christiane M-R Fauron. The physical map and organisatlon of the mitochondrial genome from the fertile cytoplasm of maize. *Nucleic acids research*, 12(24):9249–9261, 1984.

[14] Daniel B Sloan. One ring to rule them all? Genome sequencing provides new insights into the 'master circle' model of plant mitochondrial DNA structure. *New Phytologist*, 200(4):978–985, 2013.

[15] Tobias Preuten, Emilia Cincu, Jörg Fuchs, Reimo Zoschke, Karsten Liere, and Thomas Börner. Fewer genes than organelles: extremely low and variable gene copy numbers in mitochondria of somatic plant cells. *The Plant Journal*, 64(6):948–959, 2010.

[16] Maria P Arrieta-Montiel and Sally A Mackenzie. Plant mitochondrial genomes and recombination. In *Plant mitochondria*, pages 65–82. Springer, 2011.

[17] Timothy Wai, Daniella Teoli, and Eric A Shoubridge. The mitochondrial DNA genetic bottleneck results from replication of a subpopulation of genomes. *Nature genetics*, 40(12):1484, 2008.

[18] Liqin Cao, Hiroshi Shitara, Takuro Horii, Yasumitsu Nagao, Hiroshi Imai, Kuniya Abe, Takahiko Hara, Jun-Ichi Hayashi, and Hiromichi Yonekawa. The mitochondrial bottleneck occurs without reduction of mtDNA content in female mouse germ cells. *Nature genetics*, 39(3):386, 2007.

[19] Lynsey M Cree, David C Samuels, Susana Chuva de Sousa Lopes, Harsha Karur Rajasimha, Passorn Wonnapinij, Jeffrey R Mann, Hans-Henrik M Dahl, and Patrick F Chinnery. A reduction of mitochondrial DNA molecules during embryogenesis explains the rapid segregation of genotypes. *Nature genetics*, 40(2):249, 2008.

[20] Hideki Takanashi, Takayuki Ohnishi, Mirai Mogi, Takashi Okamoto, Shin-ichi Arimura, and Nobuhiro Tsutsumi. Studies of mitochondrial morphology and DNA amount in the rice egg cell. *Current genetics*, 56(1):33–41, 2010.

[21] Studies on the development and three-dimensional reconstruction of giant mitochondria and their nuclei in egg cells of *Pelargonium zonale Ait.*, author=Kuroiwa, H and Ohta, T and Kuroiwa, T, journal=Protoplasma, volume=192, number=3-4, pages=235–244, year=1996, publisher=Springer.

[22] Gayle K Lamppa and Arnold J Bendich. Changes in mitochondrial DNA levels during development of pea (*Pisum sativum L.*). *Planta*, 162(5):463–468, 1984.

[23] Dan-Yang Wang, Quan Zhang, Yang Liu, Zhi-Fu Lin, Shao-Xiang Zhang, Meng-Xiang Sun, et al. The levels of male gametic mitochondrial DNA are highly regulated in angiosperms with regard to mitochondrial inheritance. *The Plant Cell*, 22(7):2402–2416, 2010.

[24] Long Gao, Xue Guo, Xue-Qiong Liu, Li Zhang, Jilei Huang, Li Tan, Zhen Lin, Shingo Nagawa, and Dan-Yang Wang. Changes in mitochondrial DNA levels during early embryogenesis in *Torenia fournieri* and *Arabidopsis thaliana*. *The Plant Journal*, 95(5):785–795, 2018.
