# Supplementary figures and images for: Avoiding organelle mutational meltdown across eukaryotes with or without a germline bottleneck
Source: PLoS Biol. 2021 Apr 23;19(4):e3001153. doi: 10.1371/journal.pbio.3001153 (PMC8064548; doi:10.1371/journal.pbio.3001153)

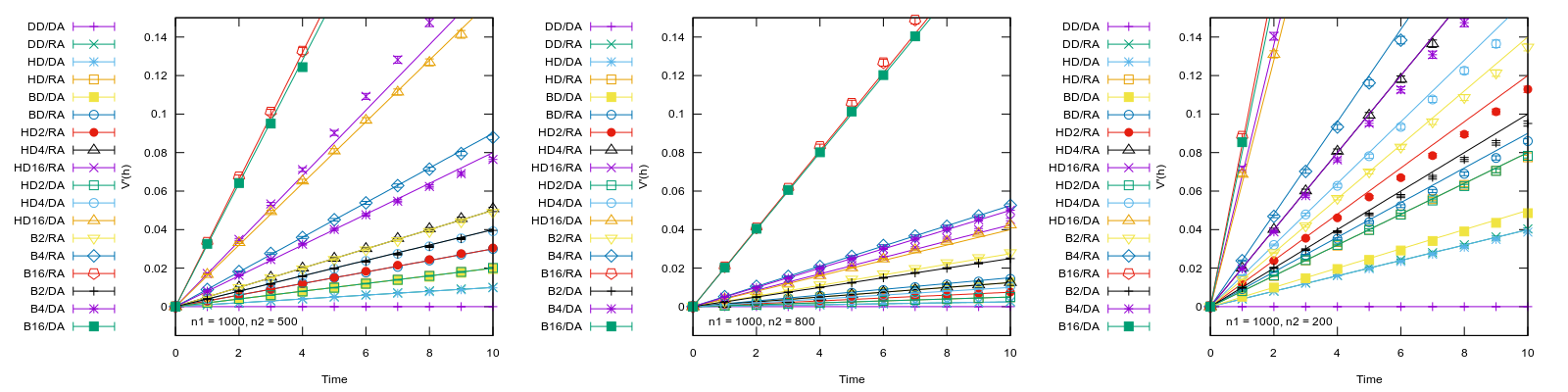

Supplement: S1 Fig — Theory and stochastic simulations (104 repeats; error bars—often small compared to the point labels—give 95% confidence intervals) of heteroplasmy level variance V′(h) as a function of time for different mechanisms. Labels give (division)/(reamplification) dynamics. Division can be deterministic (DD), hypergeometric (HD[nc]), or binomial (BD[nc]), where nc is cluster size (or individual molecules if absent). Reamplification can be deterministic (DA) or random (RA). The three panels correspond to different post-division population size n2, reflecting either halving (n2 = 500) or more budding-like divisions (lower/higher n2). (TIF) [file pbio.3001153.s002.tif]

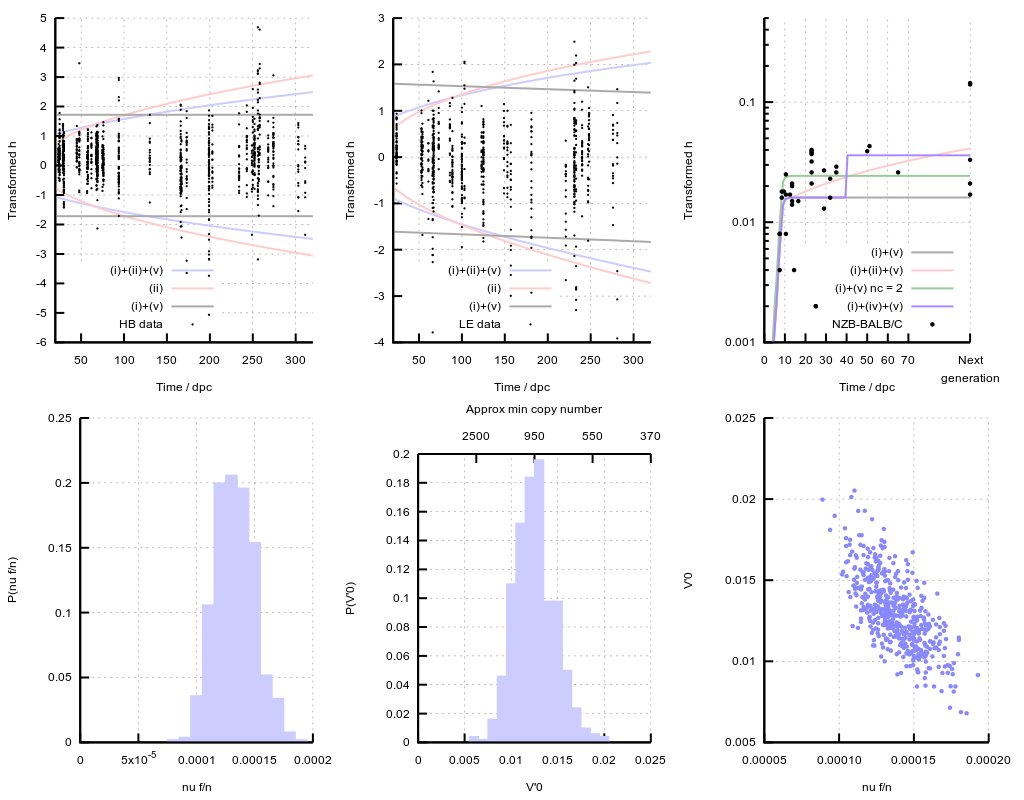

Supplement: S2 Fig — (A–C) Distributional and variance predictions, following Fig 1 C–F, for different combinations of (i) binomial cell divisions, (ii) mtDNA turnover, (iv) mtDNA subsampling, and (v) mtDNA reamplification. In each case, the different models are fitted to data. (A) Application of neutral variants of model to data from the HB model of reference [11], following Fig 1C. (B) Application of non-neutral variants of model to data from the LE model of reference [11], following Fig 1E. (C) Application of neutral variants of the model to the NZB-BALB/C model from references [37,36,18] (following reference [16]). (i)+(v) contribute early variance, of magnitude V0, during the developmental bottleneck; (ii) contributes ongoing variance increase at rate 2νf/n. (iv), allowing only a proportion of mtDNA molecules to replicate, can potentially contribute variance over different timescales; here, we illustrate it as a single discrete event during oogenesis [37], but other instances give contributions comparable to the (ii) model [16]. (D–F) Bootstrapped distributions for the (i)+(ii)+(v) model and the HB model, for (D) νf/n, (E) V0, and (F) both variables. As the copy number dynamics and timing of early mouse development have been well characterised, a given V0 value can be interpreted as a value for b, the minimum mtDNA copy number during development; we present example values on the upper horizontal axis. (F) shows that a low value from 1 variance contribution can be compensated by a high value from the other. mtDNA, mitochondrial DNA. (TIF) [file pbio.3001153.s003.tif]

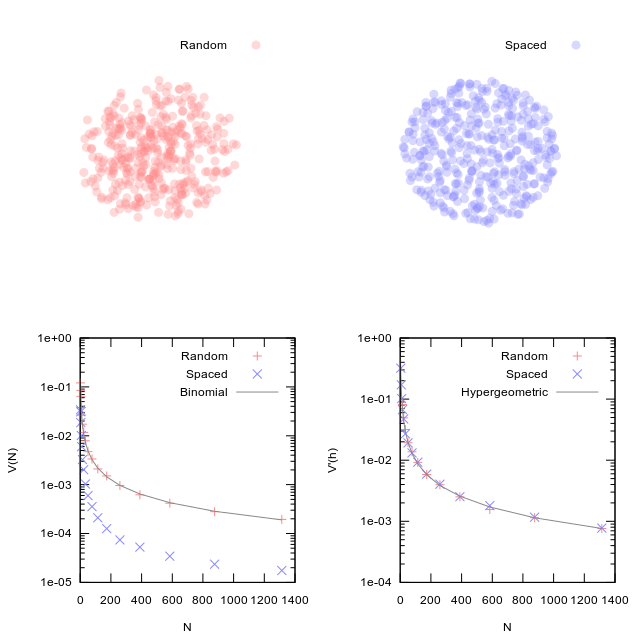

Supplement: S3 Fig — (Top) Example snapshots of the interacting and noninteracting simulations. (Bottom) Variance of copy number N and heteroplasmy level h for each case. (TIF) [file pbio.3001153.s004.tif]

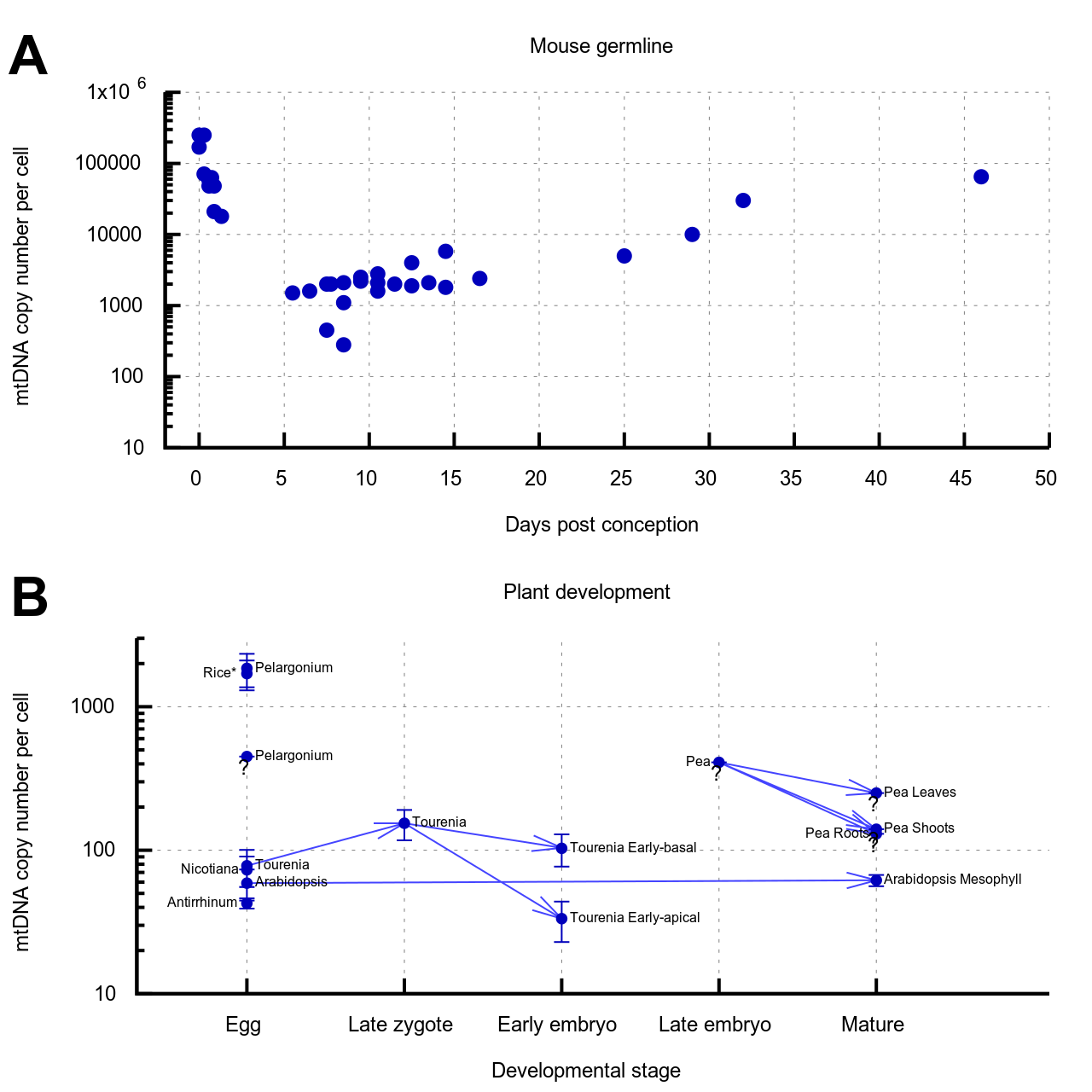

Supplement: S4 Fig — Measurements from (A) mouse models and (B) sparser measurements from different plants (see text), where arrows link observations across development in the same species from the same study. *, mtDNA copy number in rice estimated via copy number of individual mtDNA genes. Question marks denote averages for which uncertainty is not immediately available from the source publication. mtDNA, mitochondrial DNA. (TIF) [file pbio.3001153.s005.tif]

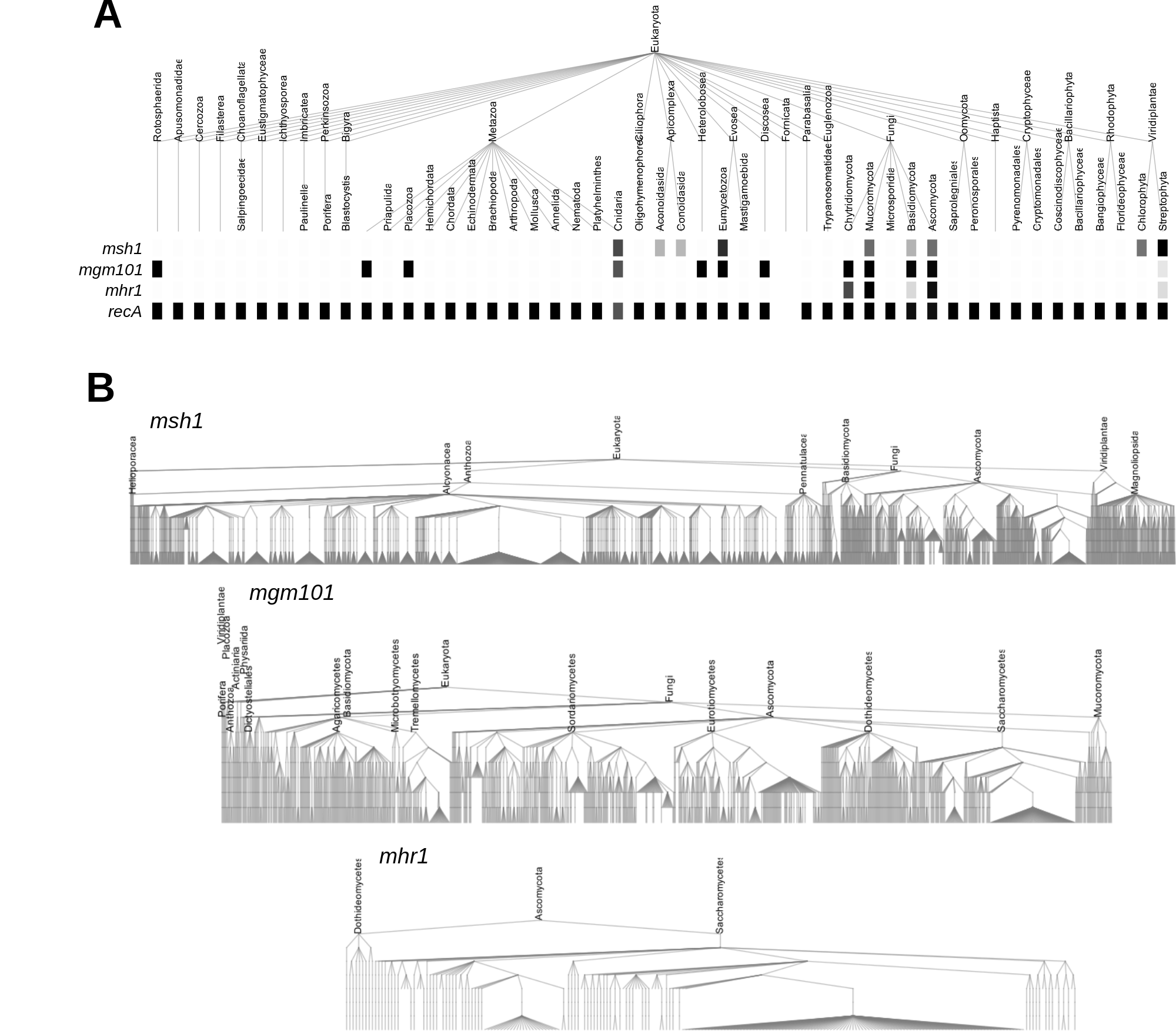

Supplement: S5 Fig — (A) Reduced taxonomic tree, corresponding to an averaging of gene presence over leaves in Fig 3. Grayscale rectangles give the proportion of leaves under each parent node that were found to contain the given gene (white, none; black, all). (B) Taxonomic trees linking BLAST hits for msh1, mgm101, and mhr1 across eukaryotes. Anthozoan species are highly sampled for msh1 because the gene is present in the mitochondrial genome and hence historically easier to characterise; the plant group in the figure reflects the vast majority of annotated plant genomes. Groups labelled include soft corals (Alcyonacea), blue corals (within Helioporacea), and sea pens (Pennatulacea). mgm101 is ubiquitous in fungi; in addition to those fungal families labelled, we also find hits in placozoans (Placozoa), sea anemones (Actiniaria), sponges (Porifera), corals (Anthozoa), slime moulds (Dictyosteliales), and other protists (for example, Physariida). mhr1 is more limited to ascomycete fungi. (TIF) [file pbio.3001153.s006.tif]
