# Supplementary material for: Avoiding organelle mutational meltdown across eukaryotes with or without a germline bottleneck
Source: PLoS Biol. 2021 Apr 23;19(4):e3001153. doi: 10.1371/journal.pbio.3001153 (PMC8064548; doi:10.1371/journal.pbio.3001153)
Supplement: Attachment — Submitted filename: reviewers-plosb.pdf [file pbio.3001153.s007.pdf]

We greatly appreciate each reviewer's time and energy in reading and commenting on our manuscript. We were delighted with the positive response to our work and greatly appreciate the reviewers' comments, which we believe have helped immensely in further strengthening our manuscript. We address individual points below.

REVIEWERS' COMMENTS:

Reviewer #1:

The genomes of mitochondria and chloroplasts are passed from generation to generation via only one of the parents, which makes organelle DNA susceptible to Muller's ratchet. The question is then, how can organisms avoid the gradual accumulation of mutations in their organelles?
The authors constructed an analytical mathematical model that investigates the effects of various mechanisms like a germline bottleneck, gene conversion, mitochondrial dynamics and cell division on the ability to generate cell-to-cell variance in the level of heteroplasmy. This variance can then be used by selection processes to prune cells carrying a high mutational load.

This work studies an important question and their mathematical model provides surprisingly simple expressions for the contributions of different biological mechanisms to cell-to-cell heteroplasmy level variance (Box 1). One of the main results is that gene conversion acts independently of organelle copy number. This explains how organisms without organelle bottleneck (like plants) can avoid mutation accumulation. This insight nicely explains and unifies different organelle structure and behaviour across different taxa.

The analytical model is complemented by stochastic simulations and analysis of different experimental data sets that corroborate and support the analytical results. I very much like this combination of approaches and have only a few minor suggestions to make.

We are very grateful for the reviewer's thorough reading and positive comments!

In Fig 1B the bars show analytical predictions and the white points results from stochastic simulations. Is that the mean of 5000 repetitions, as mentioned in line 429? This should be mentioned in the figure legend and maybe it would be useful to add 95% confidence intervals.

Thanks for pointing out this lack of clarity. These were indeed means of 5000 repeats. We have (just because it's simpler to write 10^4) increased this to 10000 and now state this in the figure caption, and have estimated confidence intervals which now appear in the plot.

In Fig 1CDE what is the meaning of 'Transformed h' and 'dpc' ?

We've expanded on these terms. dpc is days post conception, a unit of time in developmental studies. Transformed h refers to a transformation applied to heteroplasmy values to account for potentially heterogeneous initial conditions. We described this in the SI but this was too inaccessible; we have now clarified the figure caption and introduce the transformation in Methods.

In Fig S1 I wonder again about the data points for the numerical simulations. How many repetitions have been used and what is the CI ?

This simulations were carried out with the same protocol as above – they are now the means of 10000 repeats and we have plotted c.i.s.

In summary, this paper studies an important questions regarding the inheritance of organelle DNA and provides interesting and elegant answers. I therefore like to recommend it for publication after minor modifications.

Reviewer #2:

High cell-to-cell variance in heteroplasmy (frequency of deleterious mutant organismal DNAs, such as mitochondria) allows purifying selection to purge deleterious mutations, thus slowing Muller's ratchet. The most familiar mechanism employed by organisms to create such variance is a genetic bottleneck that accompanies the formation of the germ line. But many eukaryotes do not sequester a germ line early in development, which prevents the use of this mechanism. In this article, the authors investigate many proposed mechanisms that can increase cell-to-cell variance in heteroplasmy, and they incorporate all of them into a single, comprehensive genetic model that tracks the dynamics of heteroplasmy variance.

The model incorporates the following mechanisms: cell division, turnover, gene conversion, cellular subsampling, and reamplification. The authors use data from mouse models to show that their theoretical predictions match empirical measurements. Having confirmed the compatibility of the model with real-world measurements, the authors highlight some of the interesting points of their model's behavior, such as the opposite effects of organelle fission-fusion state. They point out that gene turnover increases variance when molecules are in fission state (because smaller fragments can undergo autophagy), whereas gene conversion increases variance when molecules are in fusion state (because fused molecules are available for recombination). Another model result is that for most mechanisms, variance increases more when there are few oDNA molecules (this is intuitive for the bottleneck case, since its effect results from sampling error); in contrast, gene conversion increases variance at a rate independent of copy number. From this, the authors make the prediction that in organisms that cannot resort to bottlenecks (i.e. those without early germ line sequestration) gene conversion can be important. They confirm this prediction by finding that, across the tree of life, organisms without a fixed body plan typically possess mtDNA recombination genes, which organisms with a fixed body plan lack. Further empirical evidence is provided by Arabidopsis, which has fragmented mitochondria (low conversion) -- except in the shoot apical meristem, which gives rise to the germ line, where mitochondria are fused. mtDNA recombination is also modulated in plants by surveillance genes; the authors find that, in agreement with this line of reasoning, these genes are highly expressed in the SAM.

I found this manuscript to be a highly impressive piece of work. The analytical work and predictions thereof are interesting enough on their own (for instance, the clarification of the different pathways by which the fission-fusion state of mitochondria may increase or reduce variance), but I'm particularly well-impressed with the extensive use of empirical data to validate the model (mouse models) as well as to test predictions made by the model (mtDNA recombination genes across the tree of life / the fission-fusion state of mitochondria in different tissues of Arabidopsis / the gene expression of surveillance

factors in SAM in many species of plants). I don't often read articles that so thoroughly incorporate theory and data into a satisfactory and complete story.

We are very grateful for the reviewer's positive comments, and particularly appreciate this comment on the satisfying story!

I am not an empiricist, and hence cannot fully vouch for the quality or appropriateness of the empirical work. On those points, I defer to the opinion of other reviewers/editors who may be more familiarized with the relevant literature and methods. But with regard to the areas I feel competent to judge, I have no major requests for revisions, and recommend the article for publication. My only minor requests have to do with the clarity of the exposition.

I found parts of the introduction confusing: particularly, it was hard to identify precisely which mechanisms were going to be incorporated into the model and what those mechanisms consist of. The first such mechanisms are introduced in paragraph 4: it discusses recombination and fusion. Paragraph 5 again returns to recombination, and introduces the concept of gene conversion for the first time (but the relationship between these is only explained much later, in the Methods, in line 108). The other mechanisms are only introduced later: turnover only in line 135, reamplication in line 106. It is not until the third paragraph of the Results section that we get a straightforward list of mechanisms. I think that it would be extremely helpful to clearly define all mechanisms (partitioning at cell divisions, gene conversion, turnover, subsampling, and reamplification) early on in the Introduction, maybe in bullet form, with accompanying definitions. In doing so, please be mindful of readers who may not be entirely up-to-date with terms such as reamplification, conversion, DNA turnover, or the concept of mitochondrial fission/fusion.

Thanks for this suggestion. We have taken the following steps:
1. Introduce the classes of mechanism that we will be considering in the third paragraph of the introduction;
2. Provide further detail on the specific mechanisms in the model construction section.
The reason for splitting these two responses is that several mechanisms (turnover, reamplification, subsampling) are members of the broader class of "random replication and degradation" – just with different systematic trends (respectively, constant population size, population increase, population decrease). We hope that the model section is the more appropriate place to describe this detail and the mention of the broader class in the introduction provides a suitably intuitive overview without distracting from the narrative.

I did not fully understand some parts of the procedure used when fitting the model to the mouse data. For instance, it was not clear to me why the authors present Fig. 1D (variance and bottleneck size for measured data and model prediction) for the mouse model HB but have no equivalent figure for mouse model LE.

As far as I can understand, the authors fit the model (without selection) using mechanisms (i) cell division, (ii) turnover, and (v) reamplification, to mouse model HB. The text in Fig. 1D (and in lines 169 onward) shows how the model with all three mechanisms performs better than other, simpler models. But the text doesn't discuss a similar model comparison for the LE model. Instead, the text describes a model comparison between a model with and a model without selection. Are turnover, reamplification and cell division the best predictors for LE, as they were for HB? I understand that the authors wanted to test whether their model could correctly predict that selection played a role in the LE mice -- but would the model also predict selection in the HB mice? To be clear, I am not claiming

that there is anything conceptually incorrect with the analysis, but I would prefer if the text were less synthetic and clearly justified the reasoning behind the different choices.

Several reviewer points concerned the mouse model fit. For our response to all together, please see the end of this document.

Smaller points:

The Discussion emphasizes the different mechanisms used by metazoans with early-sequestered germlines vs organisms without a fixed body plan, and the important role gene conversion plays for the latter. I think this is a fairly central take-home message, and should be included in the Author Summary (as it is in the Abstract).

We have expanded the Author Summary (we previously wrongly thought a single sentence was required) to included this core idea.

Around line 44 - I think you should cite and discuss some of the classical models that introduce the idea that bottlenecks facilitate the purging of deleterious mutations -- at least Kondrashov (1994, Genetics) and Bergstrom & Pritchard (1998, Genetics)

We have connected to these important papers and two others we became aware of during ongoing research.

Line 49 - Define Muller's ratchet and mutational meltdown

We have briefly explained the concepts in a new clause here.

Ln 292 - typo, *in shoot*

Thanks for spotting this! We have fixed it.

Reviewer #3:

This paper presents a new theory on the generation of variance in levels of heteroplasmy in organellar genomes. It is an amazing paper. I think 'tour de force' is an appropriate term here. The paper presents a new, and very clear, model. It then shows that the model fits observational data (where that data is available). It discusses a range of important implications of the model for evolutionary biology. It then performs two quite amazing empirical analyses (a comparative analysis across many taxa, and a gene expression analysis across a range of plants). I think most people would think of the work in this paper as being more suited to a series of at least three papers, but combining them together into a single concise and well-argued paper is (in my opinion) exactly the right thing to do. I enjoyed reading it enormously, for the rigour and scope of the science, the clarity of the writing, and the almost incredible concision. I have something to aspire to.

We are very grateful for these positive comments, and are delighted that the reviewer enjoyed our work! It really made our day to receive this feedback.

Having said all of that, I of course have a few comments that I hope might help to improve the paper a little. Many of them, I suspect, are due to my misunderstanding. Still, hopefully this can provide some direction in adding a few additional explanations here and there.

Rob Lanfear

Minor

Line ~42: A completely niggly point, but I don't think the ongoing presence of mtDNA diseases proves that the shifts don't remove mutants: they could be due to recurrent denovo mutations (particularly given the high mutation rates of mtDNA, and the extremely variable mutation rates among sites). Perhaps change 'can't' from 'are rarely likely to' and link it to the evidence with something like "which perhaps helps to explain"?

We agree, and have changed the text as suggested (using our own phrasing which we hope suffices to make this point!)

It wasn't all that clear to me what the authors consider to be heteroplasmies? It seems like the assumption is that heteroplasmy refers to base-level differences between organellar genomes. But there are also structural heteroplasmies which can have similar effects. The generation of these two types of heteroplasmy can be very (very) different though.
It would be useful to make explicit exactly what is being modelled.

We have clarified this in the first section describing our model setup. We tend to think of these variants as differing at bases rather than in structure, but would (and now do!) make an argument for our approach being at least partly applicable to structural variants too. Although the generation of the two types of variant are indeed very different, our model takes as its initial condition a heteroplasmic proportion that has already been generated in the cell and (as in the next point) does not consider further *de novo* mutation, many of the processes remain applicable in the case of structural variants – with the (important) exception being gene conversion, where we do not know of evidence that structural variants can “overwrite” in the same way that base variants can. We have attempted to summarise this argument in an additional discussion point linked to the reviewer’s next point.

The list of processes in the paragraph that starts on line 104 seems to omit mutation between genotypes in the model. For single-base changes this seems reasonable (and anyway, you have to start somewhere) but some structural heteroplasmies are very stable because the mutation rates (if you can call them that, perhaps 'switching rates' is better) are very high. Clarity on what types of variation are in scope for the model would help here (see previous comment).

This is exactly right – we do not consider mutational processes coupling the oDNA types (though the model framework could certainly include such processes!). This will be a target for future work. We have both clarified this in the model description and mentioned this extension in the discussion.

Line 130: I was confused about how the influence of nonzero selective differences could be accounted for 'where neither allele experiences a selective advantage'. Perhaps the authors could clarify the conditions in the ms at this point, which I assume have to do with whether selection is operating inter- or intra-cellularly?

Apologies – we phrased this confusingly. We wanted to include the no-selection assumption tightly linked to the list of results, but as currently positioned it contrasted with the claim of model generality.

The idea is that while our theoretical framework can account for selection, we first assume an absence of selection to derive the intuitive expressions listed and in Box 1. In the case of nonzero selection the expressions become more complicated – as outlined in the Methods and presented in the SI. We have rephrased to clarify.

In the numbered list starting on line 133, 'n' denotes oDNA copy number in point (i), but 'population size' in point (ii). Can you clarify? It would be nice to keep the notation consistent, and I assume that it is, such that 'n' in point (ii) should be oDNA copy number. If 'n' in point (ii) isn't oDNA copy number, can you (a) use a different symbol, and (b) explain what the population size refers to (e.g. oDNA molecules, cells, clusters, etc).

Thanks for spotting this – you are right that we used different phrases to refer to the same quantity (we think of oDNAs as cellular populations, and tend to use “copy number” and “population size” interchangeably). We have fixed this.

On line 150 I couldn't figure out why 'genetic bottleneck' was in quotes. If you think it's not really a genetic bottleneck in the traditional sense (I can see arguments both ways) perhaps just call it something like the oDNA bottleneck?

We’ve removed the quotes. One message we want to emphasise is that the genetic bottleneck is not necessarily achieved by an identical “physical bottleneck” (copy number reduction) alone – and the quotes were an attempt to draw this distinction. But we think and hope the surrounding text makes this clear.

I would like to applaud the authors for Box 1. It's so clear! As someone who doesn't find large equations intuitive to understand, the annotations make all the difference.

Thanks for this feedback! We will be employing this style in future.

Line 176: is it possible to compare (e.g. with AIC, R-squared) the NZB-BALB/c model to the current model? If not, it would be nice to explain why not. On a similar note, I would like to know the numbers behind the statement that the bottleneck size and estimate of the combined statistic vf/n 'agree well' with the analysis of the NZB-BALB/c model.

Several reviewer points concerned the mouse model fit. For our response to all together, please see the end of this document. (On this specific point, we have included an analysis of NZB-BALB/c, and additionally provided the numbers from the previous analysis of the NZB-BALB/c model for comparison).

Line 181: what is the meaning of 'direct' in 'direct quantitative demonstration'. I agree that this is a quantitative demonstration, but I don't know the difference between a 'direct' and a (presumably) 'indirect' demonstration.

This was intended by contrast with the Johnston et al. eLife paper where this “compensation” effect was inferred from theory but not directly demonstrated. But we agree this is an awkward phrasing and have just replaced with “a quantitative demonstration”.

Line 222: I had to look at ref 49 to figure out what a 'kiss and run' event was. It's a cute name, but I think the paper would be clearer if it included a parenthetical explanation of what a kiss and run event is. I note also that it's hyphenated in ref 49 but not in this ms,

and that ref 49 suggests (I didn't follow the next level of references) that the standard understanding of kiss and run events is from vesicles fusing with the cell membrane, rather than organelles (or bits of them) fusing with each other. More reasons for a parenthetical explanation I would argue.

Thanks for pointing out this lack of clarity – we have added a parenthetical as you suggest.

Line 258: I love this hypothesis. It clearly flows from the model and is well explained. I can see how it could apply to e.g. mtDNA in plants (which is nicely explained in the next section), but I had trouble reconciling it with cpDNA in plants. I wonder if the authors could comment (here, or in the paper, preferably the latter but I know how it goes with every reviewer asking for extra things in the manuscript) on this? Specifically, given the limitations that plants are thought to have, how could they potentially solve the problem of cpDNA heteroplasmy variance generation?

We would indeed anticipate the potential for gene conversion to be more limited in plastids, where a relative absence of organelle fusion means that the cellular population acts more as a set of small independent genetic 'pools' than one large interacting system. We have some analysis on this in the Supplementary Information. Our first point is that while this isolation limits the power of gene conversion to segregate ptDNA, it does not reduce it to zero – depending on plastid numbers (which are lower in, for example, the SAM than the hundreds seen in leaves), substantial segregation can still occur. We have included a short clause underlining this.

There is another point which is too speculative to include in the ms, but we include it here for discussion. There is a potential effect that could compensate for the reduced power of gene conversion – the higher n_d, or oDNA molecules per organelle (often hundreds per chloroplast), that amplifies the contribution of what organelle turnover can occur. Intuitively, this can be pictured as the degradation (or replication) of a chloroplast providing a hundredfold greater change to oDNA variance than the corresponding process occurring to a mitochondrion. We speculate that this may in part compensate for the reduction in power of gene conversion – but will explore this more in future!

I think the phylogeny in Figure 2 adds very little. It's very hard to see the relationships or the number of tips, and it's also very hard to figure out how the clades named in the tree match up with the gene tracks or the pictures below. I'd love to see something simpler here, perhaps just a tree that relates the major named clades, and some clearer delination between those clades that allows readers to see which parts of the gene tracks correspond to each clade.

The mtDNA work starting on line 261 is stunning, and frankly could be its own paper or series of papers. So please ignore this comment if it is inconvenient. As it is I think the conclusions being made (which are aknoweldged as being quite speculative) are perfectly appropriate from the analysis presented. Nevertheless, I would love to see something about the independence of the observations here. For example, a lot of the figure is devoted to showing that metazoa lack the first three genes listed, but that is really just a single evolutionary observation. There appears to be a lot more interesting stuff going on e.g. in Fungi, where sub-clades seem to be lacking all four genes. But even among the eight clades of interest I'd love to be able to better understand the extent to which the presence/absence of the genes of interest is correlated with development in an evolutionary sense. This could be done through a formal comparative analysis (overkill I think, given the scope and current length of the paper) or at least through a clearer tree in

Figure 2 that would at least allow readers to get a better feel for how correlated these traits may.

We appreciated this comment (and the positive words!) and agree with the reviewer. However, we wanted to maintain the individual species level representation in Figure 2 exactly to highlight some of the interesting diversity that the reviewer remarks upon. We have therefore proposed a possible solution. We have expanded the Supplementary Figure containing individual gene trees to also include a summary tree – Fig 2 but truncated at a higher taxonomic level, with gene presence/absence averaged over leaves falling under a given parental node. This is now linked in the Fig 2 caption so both the detail and summary tree can be observed. We agree that independence is an issue here and have added text explaining this. We further agree that a more detailed analysis would be fascinating, and are embarking upon this now!

Line 292: "occurs shoot" should be "occurs in shoot"

Thanks for spotting this – we have fixed it.

The observations, analyses, and arguments in the section on recombination genes in plants were excellent. It strikes me that these observations also seem consistent with the 'functional germline' hypothesis, i.e. that the SAM operates in a way that makes it functionally rather similar to a segregated germline in animals. (There are lots of references about the functional germline hypothesis in the paper of mine that you already cite: ref 22).

Major

In the paragraph starting on line 40 there seems to be some tension and a lack of clarity in between the general (e.g. talk of oDNA in general, and mtDNA and plastid DNA), and the specific (e.g. a lot of talk of mammals and mtDNA). I think some clarification is warranted. The previous paragraph talks very generally about the heteroplasmy level in oDNA. And this paragraph starts with a general statement about heteroplasmy level in the germline. But then almost all of the rest of the paragraph (and all but one or two references) seem to be about mammalian mtDNA. I think it would be very useful to be as explicit as possible about how major clades (e.g. plants, animals) and organelles (mitochondria in animals, mitochondria in plants, and plastids) relate to the seemingly general statements (or alternatively, and perhaps more likely, be explicit about which clades and organelles the conclusions are being drawn from, given that often the data will be pretty patchy). Of note, this issue seems to be limited to this paragraph. The rest of the paper is very explicit about which clades and organelles are the subject.

Thanks for pointing out this unconscious focus. We have edited the second paragraph to make its focus on animal mtDNA clear. We have then, combining a response to this point and Reviewer 2's request for an earlier introduction to specific mechanisms, mentioned some more general principles in the next paragraph, where we then begin to discuss differences between animals and other groups.

I find the analyses in figure 1D convincing on visual inspection, but Figures 1C and 1E much less so. Perhaps it's just that there's a lot of overplotting, making it hard to guess the true variance from the figure (or maybe I haven't understood the transformed h properly)? With that caveat though, it does look visually like a constant variance model might fit better than the model from Eq 1 where the 95% intervals increase over time. With that in mind,

I'd really like to see something more quantitative than the statement on line 171 that "the overall model captures well the time behaviour of observed single-cell heteroplasmy level distributions". Ideally this quantification would include some absolute measure of fit (e.g. of the amount of variation in the data captured by the model) as well as some comparison to sensible null (aka completely uninteresting models), and/or a purely descriptive model that shows the 95% confidence intervals in transformed h that you get directly from the data (it does seem like this might be exactly what's in Figure 1D, but I had a hard time figuring out if this is actually the case). In short, I remain a little bit less than convinced that the conclusion "the overall model captures well the time behaviour of observed single-cell heteroplasmy level distributions" is supported by the data presented. Perhaps all I need is a bit more hand-holding (e.g. if Figure 1D is already doing most of what I ask for). But even in that case I'd like to see an absolute measure of fit for 1D, like an R-squared or similar. AICs are all very well, but there will always be a model with a lowest AIC…

Several reviewer points concerned the mouse model fit. For our response to all together, please see the end of this document.

Mouse model fit
Our summary of the reviewers' points regarding this aspect of the ms are:
R2 – can we apply the same model analysis to the LE model as we do to the HB model?
R3a – can we apply the same model analysis to the NZB/BALB-c model as we do to the HB model?
R3b – can the model fit to LE and HB [we also included NZB/BALB-c in this request] be presented with more convincing/intuitive statistics / visuals?

We originally provided some additional model comparisons in Fig S2, but these were not presented in concert with intuitive statistics. We have therefore expanded Fig 1 and Fig S2 to include (we hope) the points the reviewers requested. The same analysis is applied to LE in Fig 1, where AIC and R^2 statistics are reported; R^2 statistics are also added for the analysis of NZB-BALB/C in Fig S2.

There is a statistical caveat that should be mentioned here (in addition to the comment that AIC helps account for the different complexities of these models). The sample heteroplasmy variance V^(h) [apologies for the awkward typesetting – we mean "V hat" here] of a sample of oocytes, as presented in original Fig 1D and S2B-C and now in Fig 1D, 1F, and S2C, provides an easily visible illustration of agreement between model and observation. But where individual cell data is available, the task of best-fitting the data is not the same task as best-fitting V^(h). V^(h) itself has uncertainty which increases with its absolute value, and is only a summary of an underlying distribution of points, which the theory describes. The AIC, while certainly not a flawless readout of model quality, does include an explicit likelihood function based on individual observations, which we retain for its interpretability. We also include R^2 statistics based on (log-transformed) V^(h) as requested, with a methods notes briefly mentioning this point.

To summarise – we now include R^2 statistics comparing V^(h) with theory for different neutral models for HB, different neutral and non-neutral models for LE, and different neutral models following previous literature from NZB/BALB-c. We also provide AIC values from single-cell measurements for the HB and LE cases, but not NZB/BALB-c, where we do not have single-cell measurements. R^2 and AIC agree in all cases except LE, where the R^2 is slightly lower for the case with selection (which is favoured by AIC). This is because the V^(h) comparison alone omits E^(h) behaviour, which is captured by the non-neutral

model (hence its better AIC). The R^2 statistics are not particularly high (although fairly good for HB), but this is unsurprising given the noisy nature of the V^(h) estimator.
